# Effect of leg immersion in mild warm carbonated water on skin and muscle blood flow

**DOI:** 10.14814/phy2.13859

**Published:** 2018-09-17

**Authors:** Shigehiko Ogoh, Takuro Washio, Kazuya Suzuki, Keisuke Ikeda, Takaaki Hori, Niels D. Olesen, Yoshiho Muraoka

**Affiliations:** ^1^ Department of Biomedical Engineering Toyo University Kawagoe‐Shi Saitama Japan; ^2^ Institute of Personal Health Care Products Research Kao co ltd. Tokyo Japan; ^3^ Department of Anesthesia Rigshospitalet Copenhagen Denmark; ^4^ Department of Biomedical Sciences University of Copenhagen Copenhagen Denmark; ^5^ Department of Education Meisei University Tokyo Japan

**Keywords:** Carbonated water, Doppler ultrasound, near‐infrared spectroscopy, popliteal artery

## Abstract

Leg immersion in carbonated water improves endothelial‐mediated vasodilator function and decreases arterial stiffness but the mechanism underlying this effect remains poorly defined. We hypothesized that carbonated water immersion increases muscle blood flow. To test this hypothesis, 10 men (age 21 ± 0 years; mean ± SD) underwent lower leg immersion in tap or carbonated water at 38°C. We evaluated gastrocnemius muscle oxyhemoglobin concentration and tissue oxygenation index using near‐infrared spectroscopy, skin blood flow by laser Doppler flowmetry, and popliteal artery (PA) blood flow by duplex ultrasound. Immersion in carbonated, but not tap water elevated PA (from 38 ± 14 to 83 ± 31 mL/min; *P *<* *0.001) and skin blood flow (by 779 ± 312%, *P *<* *0.001). In contrast, lower leg immersion elevated oxyhemoglobin concentration and tissue oxygenation index with no effect of carbonation (*P *=* *0.529 and *P *=* *0.495). In addition, the change in PA blood flow in response to immersion in carbonated water correlated with those of skin blood flow (*P *=* *0.005) but not oxyhemoglobin concentration (*P *=* *0.765) and tissue oxygenation index (*P *=* *0.136) while no relations was found for tap water immersion. These findings indicate that water carbonation has minimal effect on muscle blood flow. Furthermore, PA blood flow increases in response to lower leg immersion in carbonated water likely due to a large increase in skin blood flow.

## Introduction

Thermal therapy (heating) such as sauna therapy may be a therapeutic option for patients with hypertension, chronic heart failure, and coronary artery disease (Imamura et al. 2001; Kihara et al. 2002, 2009; Miyata et al. 2008). Especially, acute leg heating at 45°C for 20 min improves endothelial function and reduces oxidative stress in patients with chronic heart failure (Inoue et al. 2012). However, whole body heating may induce heat stress and increases sympathetic activity and thereby challenges the cardiovascular system (Frishman et al. 2005; Eren et al. 2009). Thus, acute leg heating may be harmful in patients with cardiovascular disease despite its beneficial effect on endothelial function. Recently, we demonstrated that acute lower leg immersion in carbonated water at 38°C improves popliteal artery (PA) flow‐mediated dilation (FMD) and decreases pulse wave velocity whereas no change was observed in response to immersion in tap water at the same temperature (Ogoh et al. 2016). These findings indicate that immersion in carbonated water improves endothelial‐mediated vasodilator function and decreases arterial stiffness while causing minimal heat stress. Thus, bathing in carbonated water could be a useful therapeutic option for improving arterial function in patients with cardiovascular disease as vascular function may be enhanced despite minimal heat stress.

The improved endothelial function observed in the lower leg in response to immersion in carbonated water may relate to changes in the cutaneous and muscle vascular bed. Immersion in carbonated water increases skin blood flow (SkBF) (Ito et al. 1989; Hartmann et al. 1997; Nishimura et al. 2002) by diffusion of CO_2_ into the subcutaneous tissue (Schnizer et al. 1985; Ito et al. 1989) and this CO_2_‐induced cutaneous vasodilation is mediated in part by nitric oxide (Fukuda et al. 1990; Papapetropoulos et al. 1997). Moreover, the improvement in FMD associates to the increase in SkBF during acute leg immersion in carbonated water at 38°C (Ogoh et al. 2016). This result is supported by the suggestion that enhanced SkBF may be important for improving microvascular vasodilator function (Green et al. 2010). On the other hand, the muscle vascular bed of the leg is larger than that of the skin and plays an important role in vascular function (Markos et al. 2013). In mice, muscle blood flow of ischemic hind limbs increases after 4‐weeks of daily immersion in carbonated water at 37°C for 10 min (Irie et al. 2005). Moreover, immersion of ischemic hind limb muscles in carbonated water enhances vascular endothelial growth factor, nitric oxide synthase phosphorylation, and cyclic guanosine monophosphate. These findings suggest that repeated leg immersion in carbonated water induces angiogenesis and thereby enhances skeletal muscle blood flow by a nitric oxide‐dependent mechanism. Taken together, it is possible that the increase in muscle, rather than skin blood flow is responsible for the improvement in endothelial function and arterial stiffness in response to leg immersion in carbonated water. However, the effect on muscle blood flow in response to lower leg immersion in carbonated water remains unknown. In the present study, we hypothesized that acute immersion in carbonated, but not tap water, at 38°C increases muscle blood flow. We evaluated PA blood flow by duplex ultrasound and gastrocnemius muscle oxyhemoglobin concentration (HbO_2_) using near‐infrared spectroscopy (NIRS) as an index of muscle blood flow (Fadel et al. 2004; Hachiya et al. 2008; Lucero et al. 2018) during acute immersion of both lower legs in carbonated and tap water at 38°C.

## Methods

### Ethical approval

All study procedures were approved by the Ethics Committee on Human Research at Toyo University, Kawagoe‐shi, Saitama, Japan (#TU2016‐020). For participation, each subject provided written informed consent according to the principles of the Declaration of Helsinki.

### Subjects characteristics

Ten men (age, 21 ± 0 years; height, 173 ± 8 cm; weight, 64 ± 9 kg; mean ± SD) participated in this study. Subjects were healthy, nonsmokers and a preparticipation questionnaire confirmed the absence of any known cardiovascular disease or risk factors and subjects receiving any medication were excluded. Subjects were instructed to abstain from caffeinated beverages and alcohol and avoid strenuous exercise for 24 h before the experiment. The experiment was performed at least 2 h after a light meal.

### Experimental Procedure

#### Leg immersion protocol

**Figure 1:**
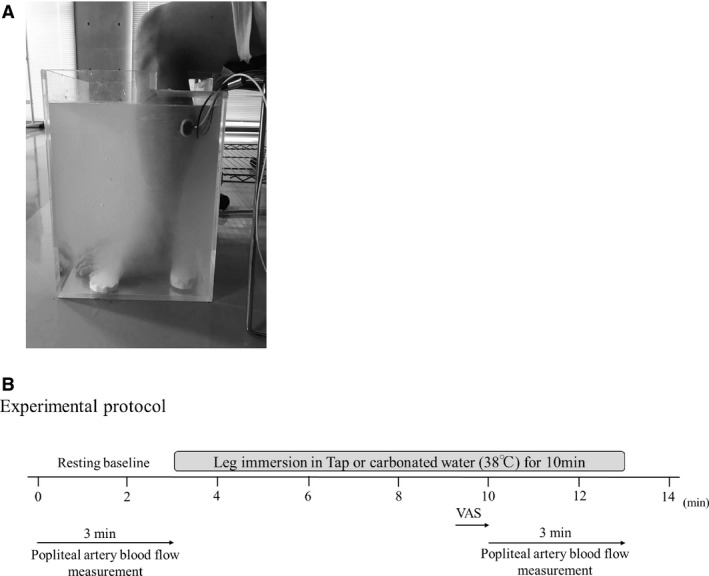
(A) Setup of the lower‐leg water immersion (A) and Experimental protocol (B).

The subjects were familiarized with the equipment and procedures before initiating the experiment. All experiments were performed in an air‐conditioned room at a constant temperature (23–24°C). The subjects sat comfortably in a chair for at least 30 min, with their knees at a 90° angle, followed by instrumentation. After a 5‐min baseline measurement, each subject in the sitting position, immersed both lower legs to 6 ± 3 cm below the popliteal fossa in tap (CO_2_ at ~2 ppm) or carbonated water (CO_2_ at 1000 ppm) at 38°C for 10 min in random order (Fig. 1). Immersion in tap and carbonated water was separated by at least 30‐min and we confirmed that all hemodynamic variables had returned to the baseline level before initiating the next protocol.

The carbonated water was color‐ and odorless and was prepared by dissolving a CO_2_ tablet comprising carbonate and fumaric acid (Kao co ltd., Tokyo, Japan) in tap water. We used a temperature of 38°C as carbonated water at that temperature improves endothelial function without causing heat stress (Ogoh et al. 2016). Each subjects was asked to rate how hot he felt using a visual analog scale (VAS_hot_) at the 7th min of immersion. VAS_hot_ ranged from 0 (not hot) to 10 (extremely hot).

### Measurements

We measured blood flow in the left PA using duplex ultrasound (Vivid‐i, GE Medical Systems, Japan) with a 13‐MHz multifrequency linear probe as previously described (Boyle et al. 2013; Teixeira et al. 2017). The ultrasound B‐mode parameters were set to optimize definition of vessel walls in a longitudinal section to evaluate PA diameter as the mean of three evaluations of systolic and diastolic diameter with mean diameter calculated as:

Mean diameter=2/3×mean diastolic diameter+1/3×mean systolic diameter

Pulsed wave Doppler determined blood velocity at an angle of insonation of 60°, with a sample volume that encompassed the entire width of the artery and PA blood flow was:

$$\text{Blood flow}=\pi \times {\left(\text{mean diameter}/2\right)}^{2}\times \text{mean blood velocity}$$

The heart rate (HR) was assessed using a lead II electrocardiograph (bedside monitor, BMS‐3400; Nihon Kohden, Japan). Mean arterial pressure (MAP), stroke volume (SV), and cardiac output (CO) were measured using finger photoplethysmography by a cuff placed on the second phalanx of the middle finger of the right hand (Finometer, Finapres Medical Systems BV, Netherlands). Vascular conductance of PA was calculated as the ratio of PA blood flow to MAP. The SkBF and skin temperature (*T*_sk_) of the immersed lower leg were measured using laser Doppler flowmetry (MoorVMS‐LDF; Moor Instruments, UK) with a laser Doppler probe (VP7a/T; Moor instruments, USA), including a thermo‐sensor attached in the midline on the left gastrocnemius muscle, 13 ± 2 cm below the popliteal fossa, at a distance of 1 mm from the skin surface in order to allow water to reach the skin (Nishimura et al. 2002). Cutaneous vascular conductance (CVC) was calculated as the ratio of SkBF to MAP. Local tissue oxygenation profiles of the gastrocnemius muscle were measured using NIRS (BOM‐L1TRW, Omega Wave, Tokyo, Japan). The NIRS probe was attached in the midline on the left gastrocnemius muscle, 13 ± 2 cm below the popliteal fossa, The NIRS apparatus applies two emitter‐receiver distances and light at 695 and 830 nm to evaluate relative change in HbO_2_ and deoxyhemoglobin concentration (HHb) at a depth of 15–30 mm as calculated according to the modified Beer–Lambert law (Delpy et al. 1988; Maki et al. 1995) albeit with a contribution of SkBF (Hirasawa et al. 2015, 2016) and myoglobin (Madsen and Secher 1999). NIRS variables were only evaluated in seven subjects because for three subjects it was difficult to evaluate NIRS variables under water. Tissue oxygenation index (TOI) was calculated as:

$$\text{TOI}={\text{HbO}}_{2}/\left({\text{HbO}}_{2}+\text{HHb}\right)\times 100$$

We continuously measured PA blood flow, HR, MAP, muscle NIRS, SkBF, and *T*_sk_. Except for the PA blood flow data, all signals were sampled at a frequency of 1 kHz using an analog‐to‐digital converter (Power Lab 16/s, ADInstruments, Australia) and stored on a computer. All variables were averaged over 3 min at the end of the baseline and during the last 3 min of tap and carbonated water immersion.

### Statistical analysis

We performed a power test to determine the required number of subjects using the change in PA blood flow in response to immersion in carbonated in three subjects in the prestudy. For the power calculation a paired *t* test (*δ *= 2; *σ *= 2; *α* level = 0.05; power = 0.8) indicated that the sample size should be *n* > 7 to obtain a significant difference. Hence, we believe that the number of subjects (*n* = 10) in this study was sufficient for this analysis. Two‐way analysis of variance for repeated measures with the factors condition (carbonated or tap water), time point (before or during immersion) and the interaction of condition and time point was performed using Student–Newman–Keuls *post hoc* tests (SPSS (11.5; SPSS, Tokyo, Japan). Pearson correlation evaluated the relation between changes in PA blood flow and SkBF, CVC, HbO_2_, or TOI during immersion in tap and carbonated water. Values are presented as mean ± SD. Statistical significance was set at *P *<* *0.05.

## Results

**Figure 2:**
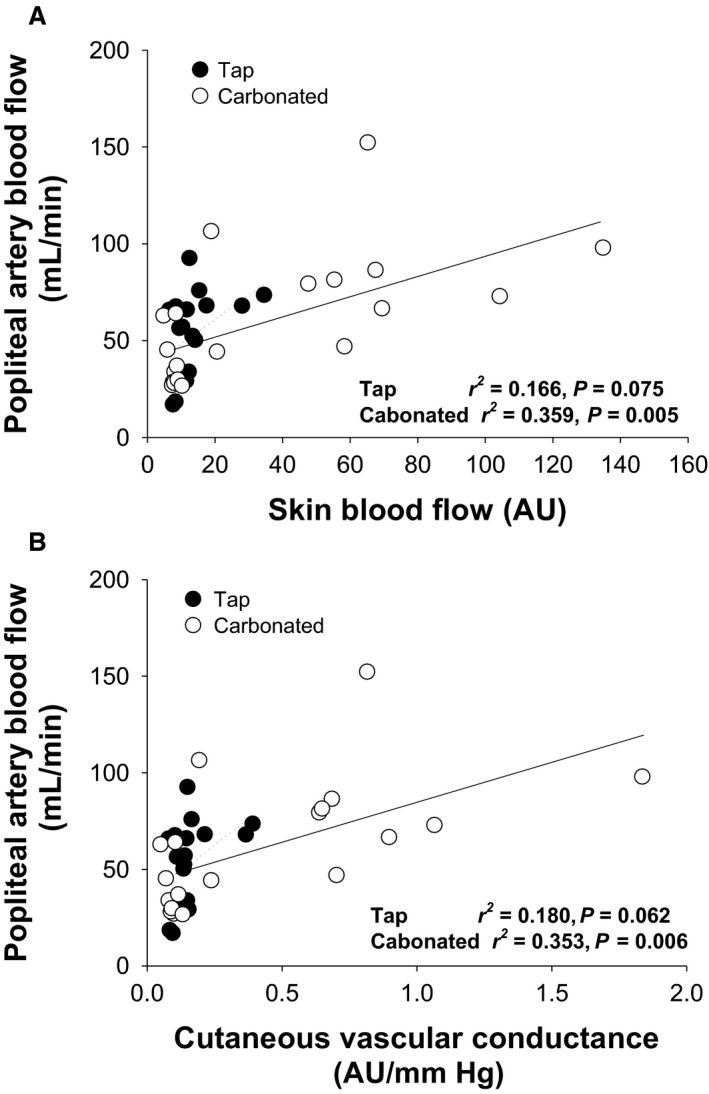
Relationship between changes in popliteal artery blood flow and skin blood flow (A) and cutaneous vascular conductance (B) during immersion in tap (●) and carbonated water (○).

**Figure 3:**
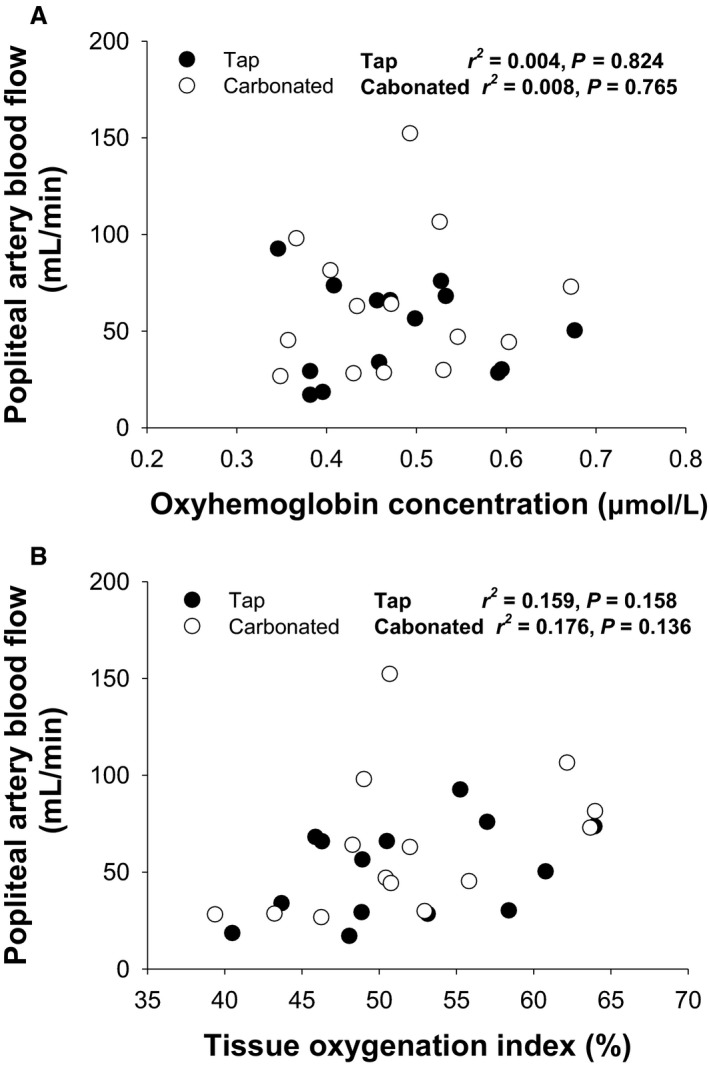
Relationship between changes in popliteal artery blood flow and muscle oxyhemoglobin concentration (A) and muscle tissue oxygenation index (B) during immersion in tap (●) and carbonated water (○).

Acute immersion of the lower legs in both tap and carbonated water at 38°C increased *T*_sk_ similarly (to 37 ± 0°C; *P *<* *0.001, Table 1). Immersion in carbonated water did not affect MAP, HR, SV, and CO while MAP increased marginally in response to immersion in tap water. Both SkBF and CVC increased by immersion in carbonated (*P *<* *0.001) but not tap water (*P *=* *0.362 and *P *=* *0.428 respectively). The VAS_hot_ was unaffected in both conditions. Acute immersion of the lower legs in carbonated and tap water did not affect PA diameter while its blood velocity increased in response to immersion in carbonated (from 3.0 ± 1.6 to 6.5 ± 3.3 cm/sec, *P* < 0.001) but not tap water. Thus, PA blood flow was increased by immersion in carbonated water and the change was larger than that in response to tap water (by 126 ± 69% vs. 36 ± 42%; *P *=* *0.005). Also, PA vascular conductance was elevated in response to immersion in carbonated but not tap water (*P* = 0.006). On the other hand, NIRS‐determined HbO_2_ (*P *=* *0.012) and TOI (*P *=* *0.004) of the gastrocnemius muscle increased during immersion in both tap and carbonated water and the change was similar for the two conditions (*P *=* *0.477 and *P *=* *0.115 respectively). The carbonated water‐induced increase in PA blood flow correlated with changes in SkBF (*P *=* *0.005, Fig. 2) and CVC (*P *=* *0.006) but not changes in HbO_2_ (*P *=* *0.765, Fig 3) and TOI (*P *=* *0.136), while the tap water‐induced increase in PA blood flow did not correlate with changes in these variables (SkBF, *P *=* *0.075; CVC, *P *=* *0.062; HbO_2_, *P *=* *0.824; TOI, *P *=* *0.158).

**Table 1:** Hemodynamic variables during lower‐leg immersion in tap and carbonated water at 38°C

	Tap water		Carbonated water		*P*‐value		
	Pre	Immersion	Pre	Immersion	Condition	Time	Interaction
Hemodynamic							
Heart rate (bpm)	73 ± 9	72 ± 9	73 ± 9	72 ± 9	0.828	0.245	0.567
Mean arterial pressure (mmHg)	85 ± 8	88 ± 10*	86 ± 9	85 ± 10	0.595	0.297	0.018
Stroke volume (mL)	82 ± 15	83 ± 15	82 ± 12	84 ± 15	0.693	0.121	0.546
Cardiac output (L/min)	5.9 ± 1.0	5.9 ± 1.0	5.9 ± 0.90	6 ± 1.10	0.658	0.612	0.260
SkBF (AU)	9.4 ± 2	16.6 ± 8	7.9 ± 2	64.3 ± 34.9* #	<0.001	<0.001	0.002
CVC (AU/mmHg)	0.11 ± 0 03	0.19 ± 0.1	0.09 ± 0.02	0.77 ± 0.46* #	<0.001	<0.001	0.002
*T*_sk_ (°C)	28 ± 1	37 ± 0	28 ± 1	37 ± 0	0.751	<0.001	0.747
VAS_hot_	4.6 ± 2.4	4.2 ± 1.7	4.0 ± 2.0	3.7 ± 2.1	0.195	0.461	1.000
NIRS (gastrocnemius muscle)							
HbO_2_ (*μ*mol/L)	0.44 ± 0.08	0.52 ± 0.1	0.43 ± 0.06	0.52 ± 0.11	0.529	0.012	0.477
HHb (*μ*mol/L)	0.47 ± 0.11	0.45 ± 0.13	0.48 ± 0.13	0.42 ± 0.13	0.503	0.004	0.058
TOI (%)	49 ± 6	54 ± 7	48 ± 6	56 ± 7	0.495	0.004	0.115
Duplex ultrasound (popliteal artery)							
Blood velocity (cm/sec)	3.6 ± 1.8	4.3 ± 1.6	3.0 ± 1.6	6.5 ± 3.3* #	0.194	<0.001	0.01
Diameter (cm)	0.53 ± 0.06	0.54 ± 0.06	0.53 ± 0.06	0.54 ± 0.06	0.912	0.285	0.426
Blood flow (mL/min)	48 ± 25	57 ± 17	38 ± 14	83 ± 31* #	0.273	<0.001	0.005
Condactance (mL/min/ mmHg)	0.58 ± 0.32	0.65 ± 0.22	0.45 ± 0.17	0.99 ± 0.40* #	0.293	<0.001	0.006

Values are means ± SD; *n* = 10 (*n* = 7 for NIRS variables). SkBF, skin blood flow; CVC, cutaneous vascular conductance; *T*_sk_, skin temperature; VAS_hot_, visual analog scale of how hot the subject felt; NIRS, Near infrared spectroscopy; HbO_2_, oxyhemoglobin concentration; HHb, deoxyhemoglobin concentration; TOI, tissue oxygenation index. *P <* *0.05 vs. preimmersion; ^#^ *P *<* *0.05 carbonated vs. tap water.

## Discussion

This study evaluated PA blood flow in addition to SkBF and muscle oxygenation of the lower leg in response to immersion in tap and carbonated water at 38°C and we hypothesized that muscle blood flow would increase during immersion in carbonated water. Immersion in tap and carbonated water increased muscle oxygenation similarly while water carbonation elevated PA blood flow and the increase in PA blood flow was correlated with the increase in SkBF but not muscle oxygenation. In contrast to our hypothesis, the results indicate that water carbonation affects SkBF but not muscle blood flow. Thus, the improved FMD in response to immersion in carbonated water (Ogoh et al. 2016) is likely due to changes in the cutaneous vasculature rather than muscle blood flow.

Immersion in carbonated water improves vascular function (Savin et al. 1995; Irie et al. 2005) but the effect on vascular disease states and the physiological mechanism remain poorly defined. To our knowledge, this is the first study to demonstrate that water carbonation does not affect muscle blood flow. Our conclusion regarding muscle blood flow depends on two findings in the present study. First, the NIRS‐determined muscle HbO_2_ and TOI as an index muscle blood flow increased similarly during leg immersion in tap and carbonated water, indicating that muscle blood flow increased regardless of carbonation. Second, water carbonation increased PA blood flow and the increase was related to the increase in SkBF and CVC whereas immersion in tap water did not affect PA blood flow, SkBF, or CVC. In addition, the change in PA blood flow during immersion in both conditions was not associated with changes in muscle HbO_2_ or TOI. These findings indicate that water carbonation selectively increases SkBF but not muscle blood flow. Thus, immersion in carbonated water may not be a useful therapy to modify muscle vasculature.

In contrast, a recent animal study (Irie et al. 2005) reported that repeated carbonated water immersion enhances angiogenesis and muscle blood flow in ischemic limbs. These conflicting results may be due to anatomical differences between humans and animals. The fascia of the calf that separates the subcutaneous and muscle tissue is thick in humans and may impede diffusion of CO_2_ into the muscle tissue in addition to its depth. Moreover, the study by Irie et al. (2005) reported the effect of repeated immersion in carbonated water for 4 weeks on muscle vasculature which may be different from the acute effect.

Local cutaneous vasodilation occurs during immersion in carbonated water (Schnizer et al. 1985; Ito et al. 1989; Hartmann et al. 1997) likely by diffusion of CO_2_ into the subcutaneous tissue through the skin whereby cutaneous vasodilation relates to an increase in nitric oxide due to extracellular acidosis (Fukuda et al. 1990; Gurevicius et al. 1995). Since muscle blood flow was not affected by water carbonation, it is unlikely that immersion in carbonated water at 38°C causes extracellular acidosis in the muscles. On the other hand, an increase in SkBF may play an important role for enhancing microvascular vasodilator function despite the small vascular bed (Green et al. 2010). In addition, leg immersion in carbonated water improved FMD of the lower leg (Ogoh et al. 2016). Therefore, the findings regarding muscle blood flow of the present study also suggest that the water carbonation‐induced improvement in vascular function relates to increases in SkBF (cutaneous vasodilation) rather than muscle blood flow. Moreover, it is surprising that an increase in blood flow to the small cutaneous vascular bed without an increase in muscle vascular bed could improve endothelial function during immersion in carbonated water.

Some studies (Heinonen et al. 2011; Neff et al. 2016; Thomas et al. 2016) have suggested that local heating increases muscle blood flow but applied lower limb heating at higher temperatures than in the present study. In addition, NIRS‐determined muscle HbO_2_ and TOI as an index muscle blood flow increased during leg immersion in regardless of carbonation. Thus, we cannot rule out the possibility that heating may alter the effect on muscle blood flow by carbonated water immersion. Taken together, immersion in carbonated water at high temperatures may be more effective for improving vascular function because both skin and muscle blood flow increases.

This study has several limitations. First, we did not directly measure muscle blood flow, but NIRS‐determined HbO_2_ reflects changes in muscle blood flow (Fadel et al. 2004; Hachiya et al. 2008; Lucero et al. 2018) albeit with an influence of SkBF (Ogoh et al. 2014; Hirasawa et al. 2015, 2016). Muscle blood flow in humans may be evaluated using positron‐emission tomography or magnetic resonance imaging (Heinonen et al. 2011), but is not feasible during water immersion. Core body temperature is important for cardiovascular function but was not evaluated to reduce subject's stress. The study included only men and it is possible that different results would have been obtained in women. Finally, as bathing in carbonated water could be beneficial for cardiovascular disease states further research should examine the long‐term effects of repeated bathing in CO_2_‐rich water as compared to normal tap water on clinically relevant parameters.

In summary, the main finding of this study was that lower leg immersion in water at 38°C elevates muscle blood flow with small effect of water carbonation. Yet, carbonation greatly increased SkBF and thus also PA blood flow increased. Hence, the improvement in vascular function in response to immersion in carbonated water is likely due to changes in the cutaneous vasculature rather than muscle blood flow. However, immersion in carbonated water at a higher temperature may improve muscle blood flow and could be more effective for improving vascular function by an effect on both the muscle and cutaneous vascular bed.

## Conflict of Interest

Keisuke Ikeda and Takaaki Hori are employees of Kao co. ltd but all authors including these authors report no conflicts.
